# Unilateral symptomatic Achilles tendinopathy has limited effects on bilateral lower limb ground reaction force asymmetries and muscular synergy attributes when walking at natural and fast speeds

**DOI:** 10.1186/s13047-022-00570-3

**Published:** 2022-09-07

**Authors:** Mathieu Lalumiere, Daniel Bourbonnais, Michel Goyette, Sarah Perrino, François Desmeules, Dany H. Gagnon

**Affiliations:** 1 Faculty of Medicine, School of Rehabilitation, Université de Montréal, P.O. Box 6128, Station Centre-Ville, Montreal, QC H3C 3J7 Canada; 2 Centre for Interdisciplinary Research in Rehabilitation of Greater Montreal (CRIR), Montreal, QC Canada; 3 Centre de recherche de l’Hôpital Maisonneuve-Rosemont (CRHMR), Montreal, QC Canada

**Keywords:** Electromyography, Gait, Locomotion, Motor control, Muscle coordination, Rehabilitation, Task performance and analysis

## Abstract

**Background:**

Achilles tendinopathy (AT) may affect ground reaction force (GRF) and muscle synergy (MS) during walking due to pain, biological integrity changes in the tendon and neuroplastic adaptations. The objective of this study was to compare GRF asymmetries and MS attributes between symptomatic and asymptomatic lower limbs (LL) during walking at natural and fast speeds in adults with unilateral AT.

**Methods:**

A convenience sample consisting of twenty-eight participants walked on an instrumented treadmill at natural (1.3 m/s) and fast (1.6 m/s) speeds. Peak GRF were measured in mediolateral, anteroposterior and vertical directions. Individualized electromyography (EMG) activation profiles were time- and amplitude-normalized for three consecutive gait cycles and MS were extracted using non-negative matrix factorization algorithms. MS were characterized by the number, composition (i.e., weighting of each muscle) and temporal profiles (i.e., duration and amplitude) of the MS extracted during walking. Paired Student’s t-tests assessed peak GRF and MS muscle weighting differences between sides whereas Pearson correlation coefficients characterized the similarities of the individualized EMG and MS activation temporal profiles within sides.

**Results:**

AT had limited effects on peak GRF asymmetries and the number, composition and temporal profiles of MS between symptomatic and asymptomatic LL while walking on a level treadmill at natural and fast speeds. In most participants, four MS with a specific set of predominantly activated muscles were extracted across natural (71 and 61%) and fast (54 and 50%) walking speeds for the symptomatic and asymptomatic side respectively. Individualized EMG activation profiles were relatively similar between sides (*r* = 0.970 to 0.999). As for MS attributes, relatively similar temporal activation profiles (*r* = 0.988 to 0.998) and muscle weightings (*p* < 0.05) were found between sides for all four MS and the most solicited muscles. Although the faster walking speed increased the number of merged MS for both sides, it did not significantly alter MS symmetry.

**Conclusion:**

Faster walking speed increased peak GRF values but had limited effects on GRF symmetries and MS attribute differences between the LL. Corticospinal neuroplastic adaptations associated with chronic unilateral AT may explain the preserved quasi-symmetric LL motor control strategy observed during natural and fast walking among adults with chronic unilateral AT.

**Supplementary Information:**

The online version contains supplementary material available at 10.1186/s13047-022-00570-3.

## Introduction

The Achilles tendon is the longest and most powerful tendon in the human body [1]. Tensile forces are transmitted through the tendon following contraction of the triceps surae muscles and enable ankle plantar flexion and related movements. Active plantar flexion is required for propulsion of the foot when generating functional movements, such as those required during walking and running [2]. To optimize the transmission of force during these functional movements, the Achilles tendon uses its potential to store and release elastic energy during the stretching and shortening phases, respectively [3]. For optimal function, a healthy tendon is crucial to withstand high tensile forces and to protect the triceps surae muscles from injury [2]. These high, repetitive and rapidly rising tensile forces transiting through the AT increase the risk of altering the biological integrity of the Achilles tendon [4]. In many cases, failure to achieve adaptive and restorative healing responses over time following Achilles tendon injury causes symptomatic and chronic Achilles tendinopathy (AT) [5–7].

The presence of localized pain, changes in the biological integrity of the Achilles tendon, and central adaptations typically characterize a symptomatic and chronic AT that can affect lower limb (LL) movement strategies during walking and running [8–11]. Runners with AT showed altered amplitude and duration of EMG activity at the lower limbs muscles during the different phases of running [12–16]. These muscular recruitment changes may also interact with changes in LL ground reaction forces (GRF) measured under the feet [11]. However, no significant differences in magnitude or timing of GRF were previously reported between adults with symptomatic AT and healthy counterparts [12, 16]. Overall, these movement strategy changes may consolidate over time and prompt cortical alterations related to central nervous system (CNS) motor planning, such as recruitment of a protective strategy with altered movements or reorganization of cortical regions [17–19].

To gain insight into the potential relationship between the above-mentioned changes in movement strategies and the CNS, a well-established hypothesis must be considered, namely that the CNS does not control each muscle individually, but instead adopts strategies that simplify the control of complex movements [20]. This hypothesis stipulates that the CNS has an organizational structure that synchronizes the amplitude, timing and duration of muscle activity to support coordinated movements [21]. In fact, the CNS initiates motor commands to select specific muscles to be activated at different intensities and in a coordinated way (i.e., motor modules or “muscle synergies” (MS)) by activating specific groups of motor neurons. These MS are thought to ease the potential complexity of the distinct neuronal activation of several individual muscles during coordinated movements.

**Fig. 1:**
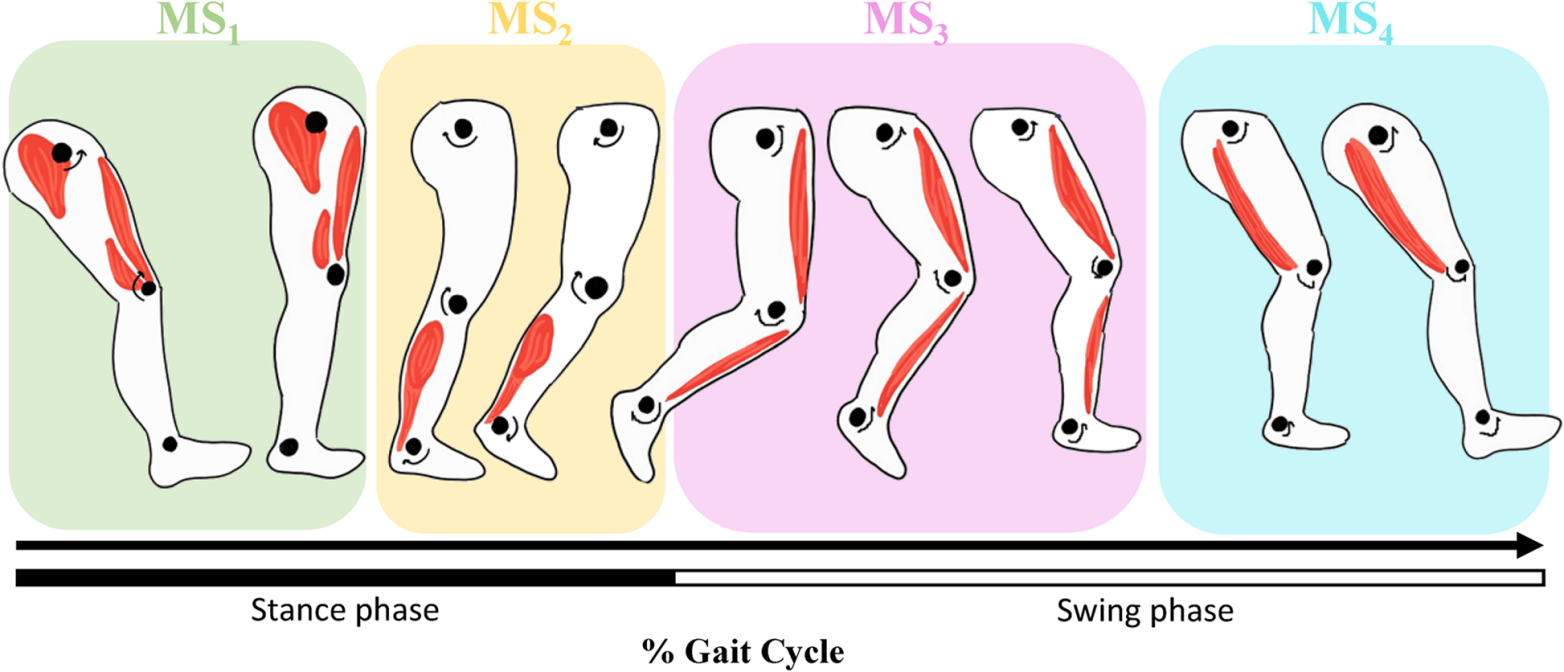
Muscular synergies identified by NNMF during walking among healthy adults

Characterizing MS in terms of the number of motor modules, composition (i.e., weighting of each muscle per motor module) and temporal profile (i.e., duration and amplitude) during different functional activities is feasible, using non-negative matrix factorization (NNMF) algorithms [22, 23]. Such a methodological approach has allowed researchers to identify four MS typically observed during walking in healthy adults [24–26]. Each of these MS is activated at distinct phases of the gait cycle, ensuring a specific biomechanical function (Fig. 1): During weight acceptance, the gluteus medius, vastus medialis and rectus femoris are activated for leg stabilization (MS_1_). During pushoff, the soleus and medial gastrocnemius are activated for forward propulsion (MS_2_). During the early swing phase, the tibialis anterior and rectus femoris are activated for swing initiation (MS_3_). During the terminal swing phase, the semitendinosus and biceps femoris are activated for leg deceleration (MS_4_).

Some MS attributes (i.e., number, composition and temporal profile) are now considered to be potential determinants and predictors of intermuscular coordination and gait performance among adults with neurological [27–29] or musculoskeletal impairments [30–32]. In adults with neurological impairments (i.e., stroke or spinal cord injury), some authors have observed that fewer MS are required to account for muscle activation during walking, suggesting a reduction in overall motor activation complexity. In adults with musculoskeletal impairments, authors have observed that adults with gluteal tendinopathy had changes in MS composition with the gluteus minimus, gluteus medius, and tensor fascia latae, notably contributing to the MS related to single leg support [33]. Authors have observed that adults with unilateral anterior cruciate ligament deficiency have altered MS activation profiles when stabilizing the knee joint with a longer activation period of the tibialis anterior, quadriceps, and hamstring muscles during stance [32]. To our knowledge, no study has characterized the effect of chronic pain and changes in the Achilles tendon on GRF and MS during walking in adults with unilateral AT.

Hence, the primary objective of this study was to compare GRF symmetries (i.e., mediolateral, posteroanterior and vertical components) and MS attribute differences (i.e., number, composition and temporal profile) between symptomatic and asymptomatic LL during walking in adults with unilateral symptomatic AT. The secondary objective was to verify if increased walking speed would reveal additional or larger differences in terms of GRF asymmetries and MS attributes between LL. For the primary objective, it was hypothesized that peak GRF in the mediolateral, posteroanterior and vertical directions would be reduced at the symptomatic LL compared to the asymptomatic LL [10]. It was also anticipated that the number of synergies would be reduced at the symptomatic LL compared to the asymptomatic LL to reflect changes in motor control complexity due to AT [34]. In addition, a change in the motor recruitment strategy of the hip (gluteus medius) and knee (vastus medialis) stabilizer muscles was anticipated and expected to translate into MS_1_ composition and temporal profile differences with the asymptomatic LL. For the secondary objective, it was hypothesized that peak GRF asymmetries would be greater at fast speed compared to natural speed whereas MS attribute differences between the asymptomatic and symptomatic LL previously described would be amplified at fast speed compared to natural speed [26].

## Methodology

### Participants

A convenience sample consisting of twenty-eight (*n* = 28) adults with unilateral, symptomatic AT of the Achilles tendon participated in this cross-sectional study. The convenience sampling technique was selected based on previous LL studies evaluating changes in muscles synergies following induced or acquired pain [30, 32, 33]. To be included, potential participants had to report unilateral, localized pain at the insertion or midportion of the Achilles tendon for more than 3 months [7], experience pain on palpation at the enthesis or midportion of the Achilles tendon, and obtain a score lower than 90 out of 100 on the Victorian Institute of Sport Assessment-Achilles Questionnaire (VISA-A) [35]. Presence of AT was confirmed by both the presence of pain upon palpation of the tendon and the observation of tendon alteration (e.g., increased tendon thickness, reduced echogenicity) during musculoskeletal ultrasound imaging. Potential participants who reported bilateral pain during sport activities, had a body mass index (BMI) greater than 30.0 kg/m^2^, reported a history of Achilles tendon rupture or repair, were diagnosed with a metabolic, neurologic or systemic inflammatory disease, or had received any type of injection at the Achilles tendon in the past year were excluded.

### Questionnaires

Basic sociodemographic data (e.g., age, sex), anthropometric data (e.g., height, mass) and AT-related information (e.g., affected side, time since onset of symptoms, etc.) were initially collected. Participants then completed two patient-reported outcome measures questionnaires: the VISA-A and the Lower Extremity Functional Scale (LEFS). The VISA-A includes eight questions targeting three dimensions: localized pain at the Achilles tendon, function in daily life and participation in sports activities [35–37]. The VISA-A questionnaire scores range from 0 to 100, with a low score indicating greater severity. The questionnaire is available in English and French and is valid, reliable, and sensitive to change with a minimal clinically important difference between 6 and 20 points [35, 38]. The LEFS questionnaire assesses function in adults with musculoskeletal disorders affecting the LL, including AT [39]. It includes 20 questions measuring the level of difficulty encountered when performing activities of daily living and sports. The LEFS has a maximum score of 80, with a high score indicating a high functional level. The LEFS, which is also available in English and French, is valid, reliable, and sensitive to change with a minimal clinically important difference of 9–12 points [38]. The VISA-A and LEFS were completed by participants either on paper or electronically via Lime Survey® in the language of their choice (i.e., French or English).

### Musculoskeletal ultrasound imaging

One trained evaluator (ML), who is an experienced physical therapist, performed all components of both the clinical and imaging evaluations. For the latest, all ultrasound images of the Achilles tendon were recorded with a HD 11XE 1.0.6 ultrasonography system (Phillips Medical Systems, Bothell, WA), using the brightness mode and a 5–12 MHz linear array transducer with a 5-cm wide footprint. A previously described standardized protocol [40] was used for image acquisition. All parameters affecting image quality remained constant for all participants during each data collection session (i.e., probe frequency set at 12 MHz; depth = 2 cm; gain = 85; unique focus zone adjusted to 0.5 cm at the Achilles tendon; neutral time gain compensation). The most painful area along the symptomatic tendon was first located by palpation before being marked on the skin and mirrored on the asymptomatic side. Three (*n* = 3) images centered on the marked site were acquired in the longitudinal plane per side.

All images were analyzed using a custom program developed using MATLAB’s Image Processing Toolbox™ (MathWorks Inc., Natick, Ma, USA) [40, 41] to extract a standardized dataset of ultrasound biomarkers (i.e., mean thickness, echogenicity, skewness, mean homogeneity, and homogeneity at 0° and 90°) [42]. Images of symptomatic tendons were expected to show increased thickness, decreased echogenicity, increased skewness, increased mean homogeneity, and increased homogeneity at 90° compared to asymptomatic tendons [42].

### Ground reaction forces

**Fig. 2:**
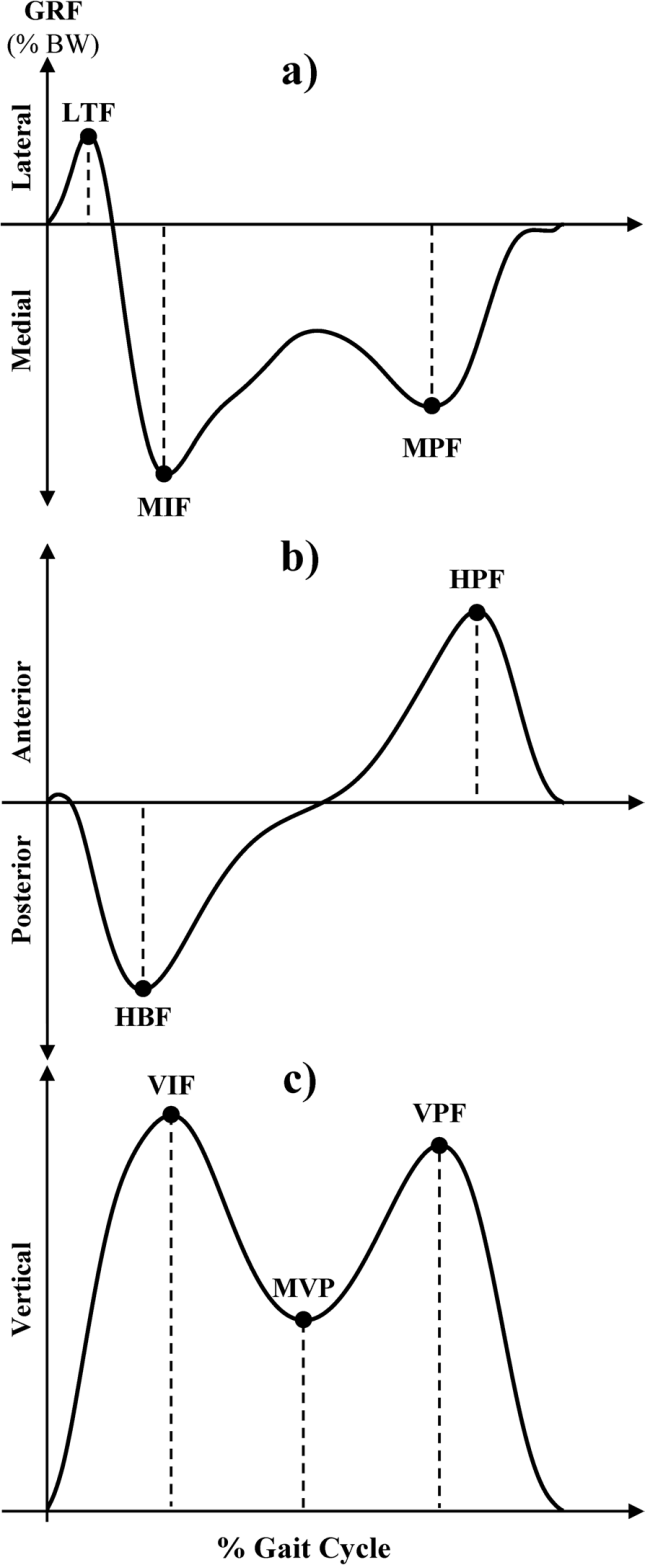
Selected ground reaction forces (GRF) in the **a** medio-lateral (GRF_ML_), **b** postero-anterior (GRF_PA_) and **c** vertical (GRF_V_) directions

GRF were recorded in mediolateral (GRF_ML_), posteroanterior (GRF_PA_) and vertical (GRF_V_) directions by a fully instrumented dual-belt treadmill with separate embedded force plates (Bertec, Columbus, USA; TM-09). Selected peak GRF_ML_, GRF_PA_ and GRF_V_ were based on curves previously described among healthy adults [43] (Fig. 2). The GRF_ML_ curve for normal walking contains one lateral and two medial peaks: the lateral thrust force (LTF) at heel contact, followed by the medial impact force (MIF) during weight acceptance, and lastly the medial propulsive force (MPF) during terminal stance. The GRF_PA_ curve for normal walking contains two peaks: the horizontal braking force (HBF) upon weight acceptance and the horizontal propulsive force (HPF) during the pushoff phase. The GRF_V_ curve for normal walking contains two peaks and a trough: the vertical impact force (VIF) during weight acceptance, the minimal vertical peak (MVP) during midstance and the vertical propulsive force (VPF) during pushoff.

### Surface electromyography

Bilateral surface EMG of the rectus femoris, vastus medialis, tibialis anterior, medial gastrocnemius, soleus, semitendinosus, biceps femoris and gluteus medius was recorded using a portable telemetric system (NORAXON USA Inc., Scottsdale, Arizona; Telemyo 900) with a frequency of 1200 Hertz (Hz). Self-adhesive surface electrodes (Ag/AgCl; Ambu_BlueSensor M) were placed in a bipolar configuration with a 1-cm inter-electrode distance over the muscle belly, perpendicular to muscle fibers orientation. Each skin site was previously shaved and cleaned with alcohol [44] in accordance with the SENIAM recommendations (refer to www.seniam.org). EMG signals were visually inspected during static voluntary contractions performed against manual resistance according to a standardized protocol [45]. The recorded EMG was filtered using a fourth-order, Butterworth, zero-lag bandpass filter with cut-off frequencies set at 20 Hz and 400 Hz. Subsequently, EMG signals were root mean squared with a centered 250 msec moving window to finally generate linear envelopes.

### Gait assessment

Participants walked on the above-described instrumented treadmill. After a 3-minute familiarization walking period on the treadmill during which participants were asked to walk at a self-selected comfortable speed, participants walked at a natural speed (1.3 m/s) for 60 s and thereafter at a fast speed (1.6 m/s) for 60 s. Since the mean self-selected comfortable speed (1.3 ± 0.1 m/s) of participants was similar to the prescribed natural speed (1.3 m/s), only the latest natural speed was considered for the analyses. A rest period was provided if required between the tasks. Pain was monitored for the symptomatic and asymptomatic tendons while walking using a 10-point visual analog scale with the ethical perspective of not increasing pain to a level greater than 5/10 in accordance with the principle of nonmaleficence [46]. EMG and GRF data were collected for the last 40 s of each trial, and an average of 3 consecutive gait cycles (two consecutive heel strikes for the same leg) were selected according to the minimal EMG variation coefficient and then used for analysis [26]. Each GRF value and EMG envelope was time-normalized (0 to 100% in 1% increments) relative to each full gait cycle, then averaged together. Each GRF value was then normalized for each participant’s body weight (BW). The amplitude of each muscle was also normalized from its peak value (0 to 1), resulting in an 8 X 101 experimental EMG (EMGexp) matrix. The mean and standard deviation (SD) of the BW-normalized GRF and amplitude-normalized EMG during the time-normalized gait cycle were calculated across subjects.

### Muscle synergies analysis

MS during walking were extracted by a NNMF algorithm using the Gamma model based on J divergence, a reliable method found to generate high coefficients of correlation and confidence levels [22] and used in previous studies to extract MS during various walking conditions [26, 47]. For each participant, the EMGexp data matrix was subjected to the NNMF algorithm. The NNMF algorithm broke down the EMGexp into two matrices and a reconstruction error (*ε*) (Eq. 1). The first matrix represents the muscle weighting (W), consisting of the contribution of each muscle (m) within each muscle synergy (s) (Eq. 2). The second matrix represents the activation timing profile (H), consisting of the muscle synergy (s) during the gait cycle for each time-normalized point (t) (Eq. 3). Agreement between EMGrec and EMGexp was then evaluated using the variance accounted for (VAF) criterion (Eq. 4).

1 $${EMG}_{exp}=W\times H+\varepsilon$$

2 $$\mathrm{W}=m\times s$$

3 $$\mathrm{H}=s\times t$$

4 $$VAF=1-\left(\sum {\left({EMG}_{exp}-{EMG}_{rec}\right)}^2/\sum {EMG_{exp}}^2\right)$$

The number of MS was determined by the least number of synergies that could explain a VAF for each muscle (*VAF*_*m*_) greater than 90% and a global *VAF* (*VAF*_*g*_) greater than 80% [48]. Whenever these criteria were reached, the reconstruction was deemed valid and the computation stopped. When the absolute difference of the coefficient of determination between the current and last pass was lower than 1 × e− 8 for 20 consecutive passes, or after 500 passes were run without convergence, the algorithm stopped. This procedure was done 20 times, and the result of the lowest reconstruction error with the lowest number of synergy modules within the validation criteria were considered adequate.

Muscle synergies were sorted out based on similarities in muscle weighting across participants and walking speeds using cosine similarity [49]. The inner product of the compared muscle synergy vectors were calculated, and the cosine angle between those synergies were measured. Sorting was performed by grouping muscle synergies based on the values of cosine similarity against a healthy reference [24–26]. The sequence of two muscle’s weightings values to be compared are viewed as vectors and the dot product of the vectors are divided by the product of their length providing an index of similarity with a maximal unitary value. Whenever the cosine similarities of W between the reference and other MS were over 0.80, MS were considered similar [50, 51]. Likewise, whenever two MS at the same walking speed were classified into the same MS group, these two synergies were considered to be merged together. The MS with the lowest cosine similarity was considered to be merged to the main MS presenting the highest correlation value.

### Statistical analysis

Descriptive statistics (i.e., mean, standard deviation (SD), proportion, range) synthesized the sociodemographics, anthropometrics, questionnaires and ultrasound-related outcomes. To describe AT-related changes at the Achilles tendon, ultrasound biomarkers (i.e., mean thickness, echogenicity, skewness and homogeneity) were compared between the asymptomatic and symptomatic LL using paired Student’s t-tests. To test the first hypothesis, GRF measures were compared using paired Student’s t-tests between the asymptomatic and symptomatic LL. Effect sizes were also computed using Hedges’ g [52] to determine the absolute magnitude of the estimates. Thus, an effect size > 0.2 was considered small, > 0.5 was considered medium, and > 0.8 was considered large [53]. Thereafter, the relative difference between the symptomatic and asymptomatic LL was computed (Eq. 5) and used to test the second hypothesis as described hereunder.

5 $$Difference\ \left(\%\right)=\frac{\left( Symptomatic- Asymptomatic\right)\ }{(Asymptomatic)}\times 100$$

To test the first hypothesis further, cosine similarities were compared between LL using paired Student’s t-tests. In addition, the weighting of each muscle within a MS was also compared using Student’s t-tests. To verify the extent to which the MS activation timing profile and EMG activation profiles were similar between LL at natural and fast speed, Pearson product–moment correlation coefficients (r) were calculated. Strength of correlation coefficients were considered negligible between 0.00 and 0.30, low between 0.30 and 0.50, moderate between 0.50 and 0.70, high between 0.70 and 0.90, and very high between 0.90 and 1.00 [54]. To test the secondary hypothesis, the difference in GRF measures between walking speed (i.e., Fast – Natural) as a percentage of BW was compared using paired t-tests between LL. To assess the change in MS attributes when going from natural to fast speed, Pearson product-moment correlation coefficients (r) of MS activation timing profiles between natural and fast speeds for both LL were calculated. Statistical analyses were carried out with SPSS v25 software and the statistical significance threshold was set at 0.05.

## Results

### Characteristics of participants

A summary of the characteristics of the participants and scores achieved on the VISA-A and LEFS questionnaires are presented in Table 1.

**Table 1:** Mean (standard deviation) characteristics of participants

Measures	Units	Mean	(SD)
Age, mean (SD)	years	42.5	(8.1)
Sex, Male/Female	number	19	/9
Height, mean (SD)	cm	1.74	(0.07)
Mass, mean (SD)	kg	78.2	(15.4)
BMI, mean (SD)	kg/m^2^	26.5	(5.0)
Symptomatic Side, Left/Right	number	18	/ 10
Time since injury	months	34.1	(30.5)
VISA-A, mean (SD)	/100	60.9	(18.2)
VISA-A, range	Min - Max	13	- 82
LEFS, mean (SD)	/100	64.7	(11.2)
LEFS, range	Min - Max	38	- 78

### Ultrasound biomarkers

A summary of Achilles tendon ultrasound biomarkers is presented in Table 2. The mean thickness revealed a significant (*p* < 0.001) and large between-side difference (g = 1.21), reaching + 29.7% for the symptomatic tendon when compared to the asymptomatic one. The echogenicity revealed a significant (*p* < 0.001) and large between-side difference (g = − 0.81), reaching − 13.9% for the symptomatic tendon when compared to the asymptomatic one. Skewness revealed a significant (*p* = 0.012) and medium between-side difference (g = 0.67), reaching a difference of + 85.5% for the symptomatic tendon compared to the asymptomatic one. The mean global homogeneity and perpendicular homogeneity (90°) respectively revealed a significant (*p* < 0.001 and *p* < 0.001) and only small between-side differences (g = 0.23 and 0.28), reaching + 2.2% to 2.9% for the symptomatic tendon compared to the asymptomatic one.

**Table 2:** Mean (standard deviation) of ultrasound variables in the longitudinal plane

	Symptomatic	Asymptomatic	Diff (%)	Effect size (g)	***p***-value*
**Musculoskeletal ultrasound biomarkers**					
Geometric					
Mean thickness (mm)	6.18 (1.24)	4.76 (1.06)	29.7	1.21	< 0.001*
Composition					
Echogenicity (/255)	66.82 (11.43)	77.62 (14.73)	−13.9	−0.81	< 0.001*
Skewness	0.320 (0.250)	0.173 (0.178)	85.5	0.67	0.012*
Texture					
Mean homogeneity	0.691 (0.064)	0.676 (0.064)	2.2	0.23	0.001*
Homogeneity at 0°	0.778 (0.062)	0.775 (0.060)	0.4	0.05	0.524
Homogeneity at 90°	0.665 (0.067)	0.646 (0.069)	2.9	0.28	< 0.001*

* Paired Student’s t-tests statistically significant at a level of *p* < 0.05

### Ground reaction forces

**Fig. 3:**
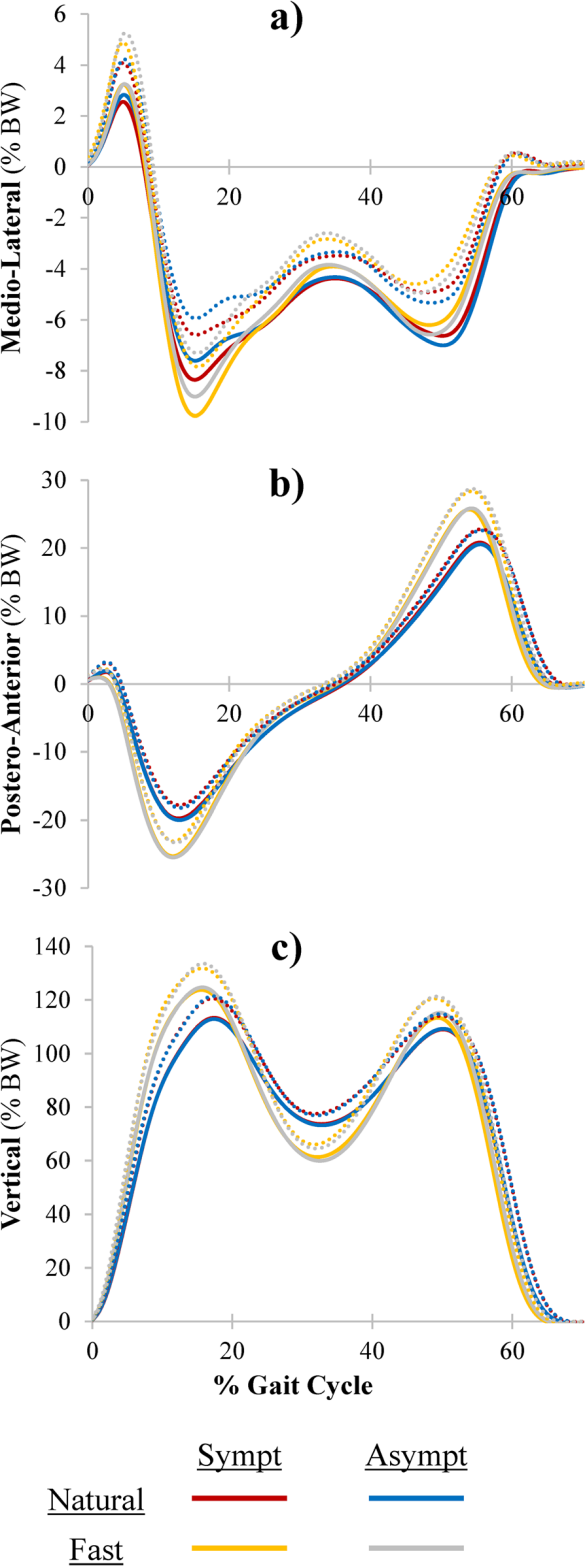
Group average (full line) and average + 1SD (dotted lines) of GRF in the **a** medio-lateral, **b** postero-anterior and **c** vertical directions for the symptomatic (Sympt) and Asymptomatic (Asympt) sides at natural and fast walking speeds

During walking at natural speed (1.3 m/s), the mean stride length was 1.39 ± 0.08 m and cadence was 56.1 ± 3.1 strides/minute for both the symptomatic and asymptomatic sides. During walking at fast speed (1.6 m/s), the mean stride length was 1.59 ± 0.09 m and cadence was 60.5 ± 3.5 strides/minute for both the symptomatic and asymptomatic sides. A summary of the mean and standard deviation (SD) of GRF_ML_, GRF_PA_ and GRF_V_ during the gait cycle and the selected peak GRF for the asymptomatic and symptomatic LL during natural and fast speed walking are presented in Table 3 and Fig. 3, respectively. Among the peak GRF, only the medial propulsive force during natural and fast walking speeds respectively revealed a significant (*p* < 0.001 and *p* = 0.022) and small between-side difference (g = 0.23 and g = 0.23), reaching − 5.46% and − 5.49% difference for the symptomatic LL compared to the asymptomatic one. Peak GRF differences between natural and fast speed were similar for the symptomatic tendon versus the asymptomatic one.

**Table 3:** Mean (standard deviation) kinetic variables

	Symptomatic		Asymptomatic		Diff (%)	Effect size (g)	***p***-value*
**Medio-Lateral GRF**							
Lateral Thrust Force (LTF)							
Natural (% of BW)	2.68	(1.46)	2.95	(1.31)	−9.24	0.19	0.211
Fast (% of BW)	3.31	(1.58)	3.53	(1.68)	−6.15	0.13	0.413
Difference (% of BW)	0.63	(0.82)	0.57	(0.87)	9.8	−0.07	0.795
Medial Braking Force (MBF)							
Natural (% of BW)	8.57	(1.62)	7.99	(1.62)	7.21	−0.35	0.024*
Fast (% of BW)	9.97	(1.66)	9.26	(1.66)	7.67	−0.42	0.003*
Difference (% of BW)	1.40	(1.34)	1.27	(1.34)	10.6	0.10	0.522
Medial Propulsive Force (MPF)							
Natural (% of BW)	6.80	(1.75)	7.19	(1.65)	−5.46	0.23	0.001*
Fast (% of BW)	6.47	(1.63)	6.85	(1.58)	−5.49	0.23	0.022*
Difference (% of BW)	−0.33	(1.02)	−0.34	(1.17)	−5.0	0.02	0.897
**Postero-Anterior GRF**							
Horizontal Breaking Force (HBF)							
Natural (% of BW)	19.97	(1.89)	20.26	(1.73)	−1.43	0.16	0.421
Fast (% of BW)	25.59	(2.27)	25.73	(2.20)	−0.55	0.06	0.800
Difference (% of BW)	5.64	(1.92)	5.49	(1.60)	2.7	−0.08	0.722
Horizontal Propulsive Force (HPF)							
Natural (% of BW)	21.10	(1.95)	20.91	(1.95)	0.91	−0.10	0.487
Fast (% of BW)	26.03	(2.76)	26.33	(2.76)	−1.12	0.11	0.389
Difference (% of BW)	4.96	(1.66)	5.38	(1.66)	−7.9	0.25	0.126
**Vertical GRF**							
Vertical Impact Force (VIF)							
Natural (% of BW)	114.34	(6.88)	114.00	(7.99)	0.30	−0.04	0.670
Fast (% of BW)	125.42	(8.06)	125.83	(8.47)	−0.33	0.05	0.684
Difference (% of BW)	11.09	(4.17)	11.84	(3.69)	−6.3	0.19	0.403
Minimal Vertical Peak (MIP)							
Natural (% of BW)	73.58	(4.80)	73.12	(4.35)	0.63	−0.10	0.388
Fast (% of BW)	62.03	(7.67)	59.63	(6.46)	4.02	−0.33	0.220
Difference (% of BW)	−11.55	(8.37)	−13.49	(8.37)	−14.3	−0.23	0.299
Vertical Propulsive Force (VPF)							
Natural (% of BW)	110.13	(4.72)	110.36	(4.92)	−0.21	0.05	0.752
Fast (% of BW)	115.17	(6.42)	116.46	(4.79)	−1.11	0.22	0.234
Difference (% BW)	5.05	(4.64)	6.10	(2.87)	−17.2	0.27	0.235

*Paired Student’s t-tests statistically significant at a level of *p* < 0.05, *GRF* Ground Reaction Forces. g: Hedges’ g

### Number of muscle synergies and cosine similarity

**Fig. 4:**
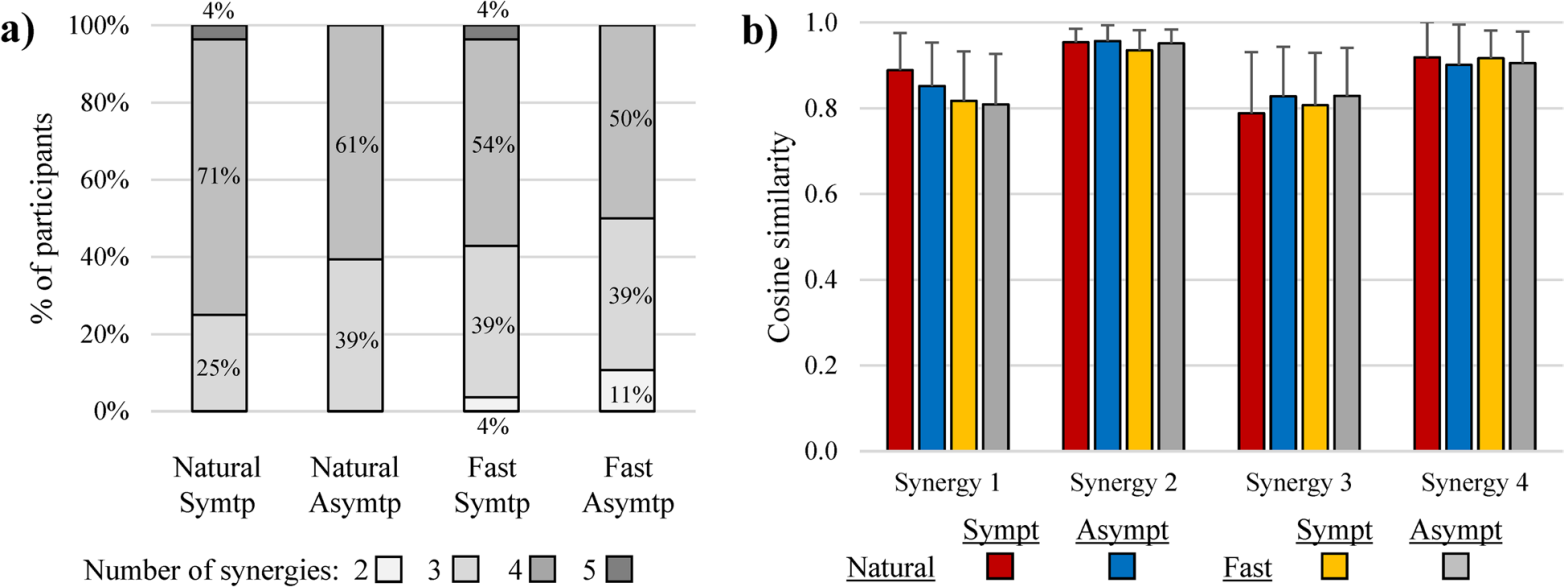
**a** Number of synergies is determined by the VAF (variance accounted for) criterion for each walking condition. **b** Cosine similarity values (r) for the weightings of each muscle synergy

Two to five MS were extracted to characterize gait using NNMF for all eight muscles for each LL (Table 4 & Fig. 4). Overall, four MS with a specific set of predominantly activated muscles (Fig. 1) were extracted among the majority of participants during natural (71% and 61%) and fast (54% and 50%) walking speeds respectively, for the symptomatic and asymptomatic LL. Specifically, MS_1_ was identified in most participants on the symptomatic LL at natural speed (93%) but in fewer participants at fast speed (86%). MS_1_ was also identified in most participants on the asymptomatic LL at natural speed (79%) but in fewer participants at fast speed (75%). MS_2_ was identified in all participants (100%) across all conditions. MS_3_ was identified in most participants on the symptomatic LL at natural speed (82%) but in fewer participants at fast speed (75%). MS_3_ was also identified in most participants on the asymptomatic LL at natural speed (93%) but in fewer participants at fast speed (75%). MS4 was identified in all participants on the symptomatic LL at natural speed (100%) but in fewer participants at fast speed (93%). MS_4_ was also identified in most participants on the asymptomatic LL at natural speed (89%) and at fast speed (89%). Hence, the number of merged synergies was higher with the asymptomatic LL compared to the symptomatic LL, with this phenomenon being accentuated further by an increase in walking speed. Additional analysis revealed that MS_1_ and MS_3_ merged more often into other synergies than MS_2_ and MS_4_. This is also confirmed by the cosine similarity values in which MS_1_ and MS_3_ have generally lower values than MS_2_ and MS_4_ (Fig. 4b). Also, the cosine similarity values were comparable (*p* > 0.05) between LL during natural and fast walking speeds for each MS (Fig. 4b).

**Table 4:** Muscle synergies detected and merged among walking conditions

		Natural (1.3 m/s)								Fast (1.6 m/s)							
		Symptomatic				Asymptomatic				Symptomatic				Asymptomatic			
		Synergy				Synergy				Synergy				Synergy			
Participant		C1	C2	C3	C4	C1	C2	C3	C4	C1	C2	C3	C4	C1	C2	C3	C4
1		✓	✓	✓	✓	✓	✓	✓	✓	✓	✓	✓	✓	✓	✓	✓	✓
2		✓	✓	✓	✓	✓	✓	✓	✓	✓	✓	✓	✓	✓	✓	✓	✓
3		✓	✓	∼C1	✓	∼C4	✓	✓	✓	∼C4	✓	✓	✓	∼C4	✓	✓	✓
4		✓	✓	✓	✓	✓	✓	✓	✓	✓	✓	✓	✓	✓	✓	✓	✓
5		✓	✓	✓	✓	✓	✓	✓	✓	✓	✓	✓	✓	✓	✓	✓	✓
6		✓	✓	✓	✓	✓	✓	✓	✓	✓	✓	✓	✓	✓	✓	∼C4	✓
7		✓	✓	∼C4	✓	✓	✓	✓	✓	✓	✓	✓	✓	✓	✓	✓	∼C3
8		✓	✓	✓	✓	✓	✓	✓	✓	✓	✓	✓	✓	✓	✓	✓	✓
9		✓	✓	✓	✓	✓	✓	✓	✓	✓	✓	∼C1	✓	✓	✓	✓	✓
10		✓	✓	✓	✓	✓	✓	✓	✓	✓	✓	∼C1	✓	✓	✓	✓	✓
11		✓	✓	∼C4	✓	✓	✓	∼C4	✓	✓	✓	∼C4	✓	∼C4	✓	∼C4	✓
12		✓	✓	✓	✓	✓	✓	✓	✓	✓	✓	✓	✓	✓	✓	✓	✓
13		✓	✓	✓	✓	✓	✓	✓	✓	∼C3	✓	✓	✓	✓	✓	✓	✓
14		✓	✓	✓	✓	✓	✓	✓	∼C3	✓	✓	✓	✓	✓	✓	✓	∼C3
15		✓	✓	✓	✓	✓	✓	✓	∼C1	✓	✓	✓	∼C1	✓	✓	✓	✓
16		✓	✓	✓	✓	✓	✓	✓	✓	✓	✓	✓	✓	✓	✓	✓	✓
17		✓	✓	✓	✓	✓	✓	∼C4	✓	✓	✓	✓	✓	✓	✓	∼C1	✓
18		✓	✓	✓	✓	✓	✓	✓	✓	✓	✓	✓	✓	✓	✓	✓	∼C3
19		✓	✓	✓	✓	∼C4	✓	✓	✓	∼C4	✓	✓	✓	∼C4	✓	✓	✓
20		∼C3	✓	✓	✓	∼C3	✓	✓	✓	✓	✓	✓	✓	∼C3	✓	✓	✓
21		✓	✓	✓	✓	∼C3	✓	✓	✓	✓	✓	∼C4	✓	∼C4	✓	∼C4	✓
22		∼C3	✓	✓	✓	✓	✓	✓	✓	∼C4	✓	∼C4	✓	∼C4	✓	∼C4	✓
23		✓	✓	✓	✓	✓	✓	✓	✓	✓	✓	∼C1	✓	✓	✓	✓	✓
24		✓	✓	∼C1	✓	∼C3	✓	✓	✓	✓	✓	✓	✓	✓	✓	∼C1	✓
25		✓	✓	✓	✓	✓	✓	✓	✓	✓	✓	✓	✓	✓	✓	✓	✓
26		✓	✓	∼C4	✓	✓	✓	✓	✓	✓	✓	✓	∼C3	✓	✓	✓	✓
27		✓	✓	✓	✓	∼C4	✓	✓	✓	✓	✓	✓	✓	∼C4	✓	✓	✓
28		✓	✓	✓	✓	✓	✓	✓	∼C1	✓	✓	∼C1	✓	✓	✓	∼C4	✓
Synergies merged	(nb)	2	0	5	0	6	0	2	3	4	0	7	2	7	0	7	3
	(%)	7%	0%	18%	0%	21%	0%	7%	11%	14%	0%	25%	7%	25%	0%	25%	11%
**Total**	(nb) **(%)**	7/112 **6.3%**				11/112 **9.8%**				13/112 **11.6%**				17/112 **15.2%**			

✓ = main synergy detected, ∼ CX = synergy merged with synergy number X

### Muscle synergies activation timing profile

**Fig. 5:**
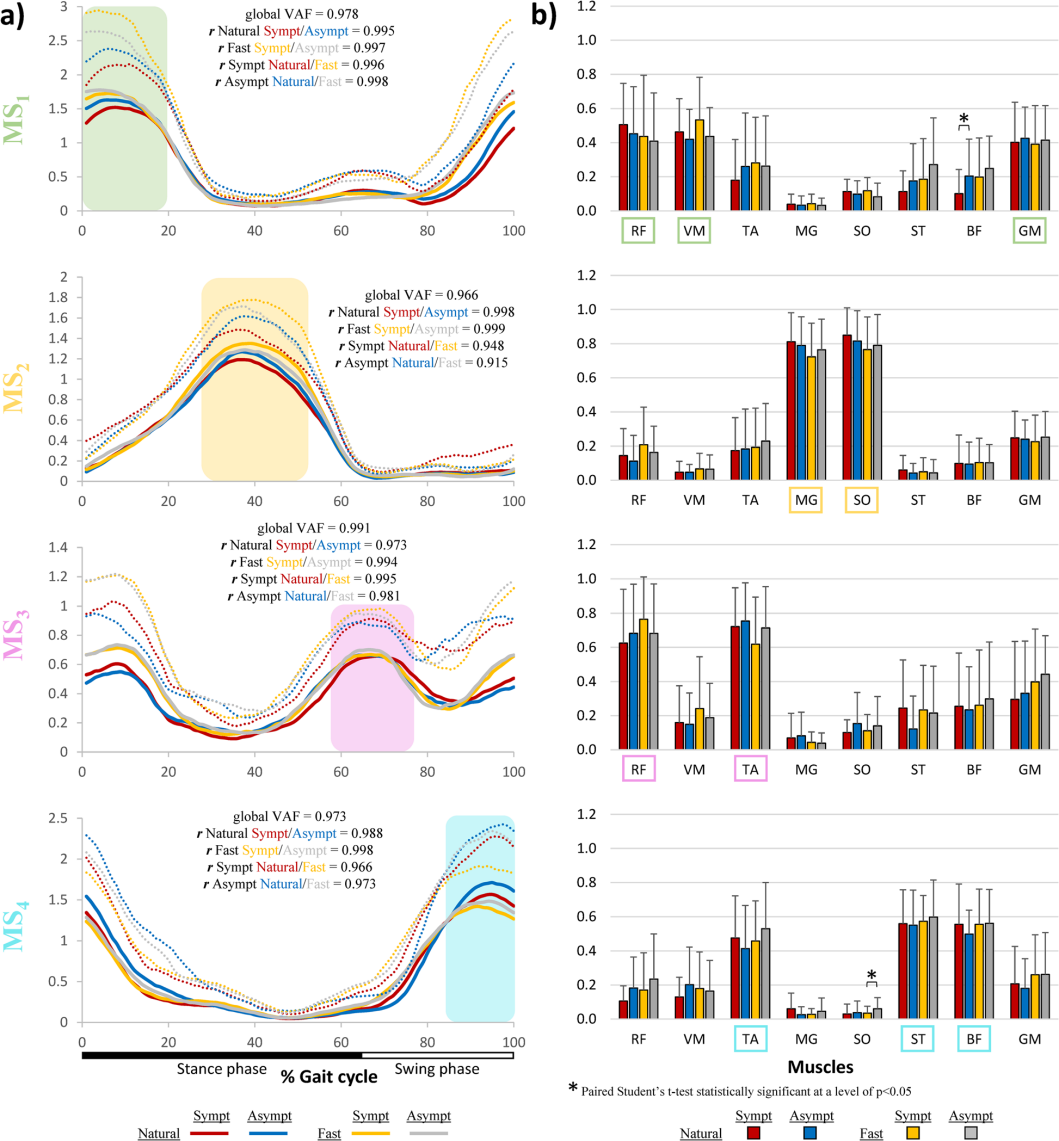
Group average (*n* = 28) for each of the four muscle synergies at natural and fast walking speeds. **a** Activation timing profiles for each synergy over the gait cycle with corresponding global VAF (variance accounted for). Group average (full line) and average + 1SD (dotted lines). **b** Muscle weightings average and SD of the four synergies. RF = rectus femoris, VM = vastus medialis, TA = tibialis anterior, MG = medial gastrocnemius, SO = soleus, ST = semitendinosus, BF = biceps femoris, GM = gluteus medius

Activation timing profiles, demonstrating how activation of each MS (i.e., MS_1_ to MS_4_) varies over the gait cycle, are shown in Fig. 5a. For each of the four main MS, the global VAF and the Pearson coefficient of correlation (***r***) between the symptomatic and asymptomatic LL for the natural and fast speed were marked on each graph. In general, the activation timing profiles for all conditions were remarkably similar for all four MS. MS_1_ confirms that the activation of the vastus medialis and gluteus medius *predominantly contributes to the synergistic* pattern observed between 0% and 20% of the gait cycle. MS_2_ confirms the activation of the soleus and medial gastrocnemius between 30% and 50% of the gait cycle. MS_3_ confirms the activation of the tibialis anterior, vastus medialis and rectus femoris with two peaks of activity occurring at the beginning and at approximately 60% to 75% of the gait cycle. MS_4_ confirms the activation of the tibialis anterior, semitendinosus and biceps femoris between 85% and 100% of the gait cycle. The global VAS was equal to 0.978, 0.966, 0.991 and 0.973 for MS_1_, MS_2_, MS_3_ and MS_4_, respectively. Correlation between the activation profiles of the asymptomatic and symptomatic LL were very high (***r*** > 0.973) in all comparisons.

### Muscle weightings

For each MS selected, muscle weightings were calculated to indicate the strength of representation of each muscle among each LL and condition (Fig. 5b). When comparing the muscle weighting between the LL, only two comparisons were found to be significantly different. For MS_1_, the muscle weighting of the biceps femoris was found to be significantly lower for the symptomatic LL (*p* = 0.008) compared to the asymptomatic LL. For MS_4_, the muscle weighting of the soleus was found to be lower for the symptomatic side (*p* = 0.03) when compared to the asymptomatic LL.

### Individualized EMG activation profiles

**Fig. 6:**
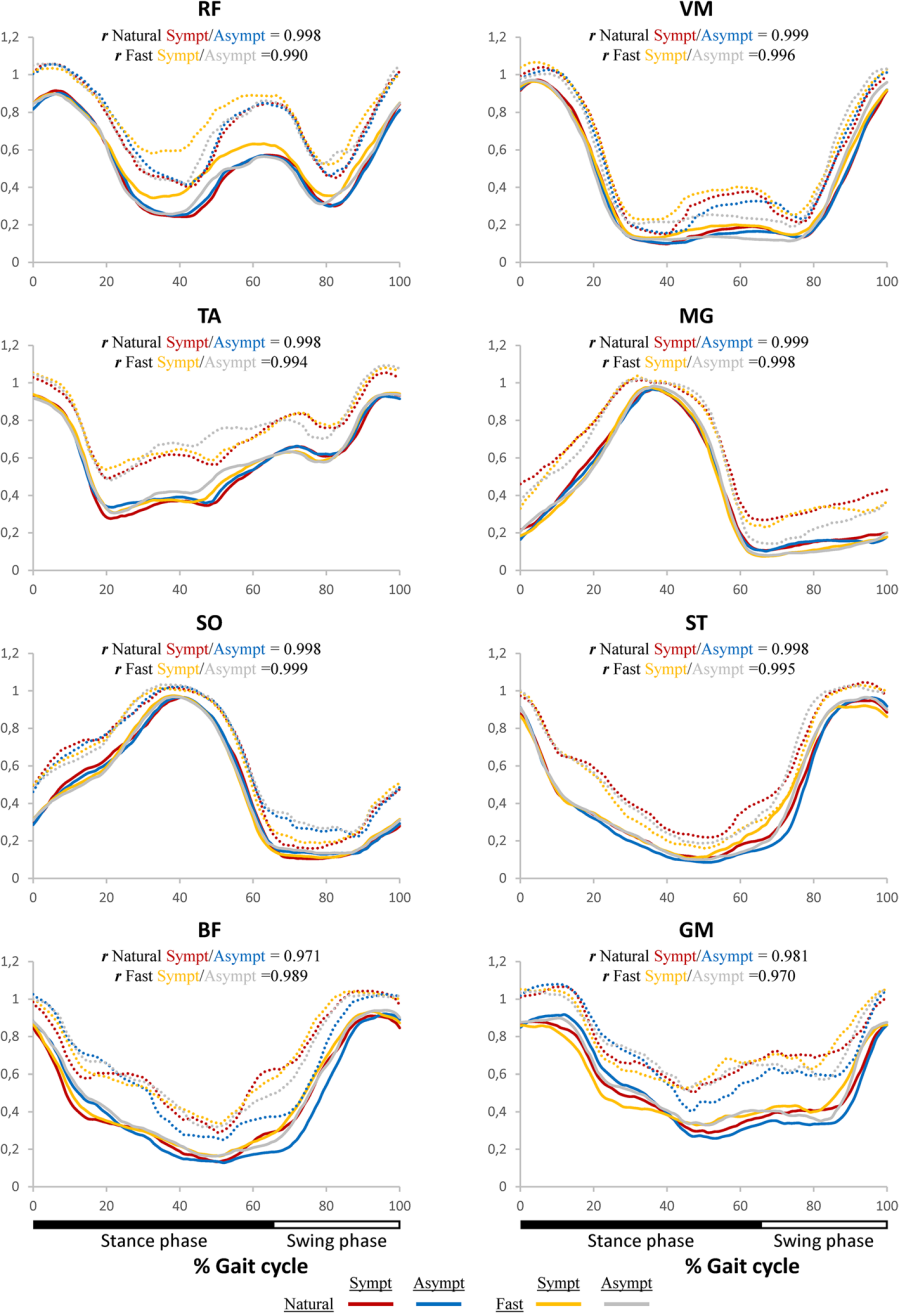
Individual EMG activation profiles over the gait cycle. Each muscle activity was normalized by maximum activation across each walking speed. Group average (full line) and average + 1SD (dotted lines)

Individualized experimental EMG activation profiles were illustrated to demonstrate the muscle activation pattern of each muscle over the gait cycle (Fig. 6). For each individualized EMG profile, the correlation between the symptomatic and asymptomatic LL for natural and fast speeds were marked on the graph. The correlation between the asymptomatic and symptomatic LL was found to be very high (***r*** > 0.970) for all comparisons.

## Discussion

The effects of unilateral AT pain and Achilles tendon integrity changes on GRF asymmetries and MS attributes during walking were investigated. Overall, the results revealed that the presence of AT had limited effects on bilateral GRF asymmetries, muscle weightings and MS activation profiles between the symptomatic and asymptomatic LL during walking at natural and fast speeds. In most participants, four MS (i.e., MS_1_ to MS_4_) were sufficient to explain the majority of the VAF (i.e., > 90% of the VAF for each muscle and > 80% of the global VAF). For all conditions, MS demonstrated relatively similar temporal activation profiles and muscle weightings to those reported in previous studies among healthy adults [25, 34, 55], with very few key differences. Increasing the walking speed from a natural pace (1.3 m/s) to a fast pace (1.6 m/s) increased some peak GRF values and increased the number of merged MS for both LL, but neither significantly altered MS temporal activation profiles nor muscle weighting between the symptomatic and asymptomatic LL.

### Limited effects on bilateral LL GRF asymmetries and MS attributes

The primary objective of this study was to compare GRF symmetries and MS attributes between the asymptomatic and symptomatic LL during walking in adults with unilateral symptomatic AT. The results partially supported the first hypothesis, which stated that peak GRF would be reduced at the symptomatic LL compared to the asymptomatic LL. First, only the two GRF_ML_ peaks were significantly different between the symptomatic and asymptomatic LL at natural and fast speeds. An increase in the GRF_ML_ peak during midstance on the symptomatic side may be explained, in part, by an increase in vastus medialis and medial gastrocnemius muscle activity to promote medial braking force [56]. A decrease in peak GRF_ML_ during terminal stance on the symptomatic side may also be explained, in part, by a reduction in hip adductor and medial gastrocnemius contractions to lower medial propulsive forces [56]. Because the absolute medial propulsive force differences between LL were ultimately very small (< 0.5% BW), it is also plausible that these potential muscle contraction differences during walking could not be detected or that they predominantly related to measurement errors associated with the treadmill or EMG recording system or both. Peak GRF in the postero-anterior and vertical directions were similar between LL at natural and fast walking speeds, which can be explained by the similar muscle activation patterns observed among all eight muscles analyzed [57].

Contrary to our hypothesis for MS, no unilateral change in the motor recruitment strategy of the hip or knee muscle stabilizers was observed during the support phase (i.e., MS_1_) for the symptomatic LL. Likewise, no motor recruitment strategy difference was observed at the ankle during the pushoff phase (i.e., MS_2_) for the symptomatic LL. Such an adaptation was anticipated to reduce the tensile force transiting through the symptomatic Achilles tendon and ultimately decrease the peak GRF_V_. Still contrary to our hypothesis, the number of merged synergies was slightly higher at the asymptomatic LL than the symptomatic one. Considering the bilateral nature of walking and the required adjustments of the CNS, these results may highlight potential motor control adaptations of the asymptomatic LL over time (i.e., chronic AT) to preserve symmetry during walking. As such, it was previously suggested that following a unilateral chronic musculoskeletal injury, neuroplastic adaptations and their effects on the CNS can explain changes in sensory and motor cortical representation, resulting in bilateral perceptual changes of body image and motor control [18]. Similarly, a relatively recent systematic review confirms the presence of sensory and motor alterations on the non-injured side of adults with unilateral tendon pain and related disability [58]. These central adaptations add to the peripheral adaptations highlighted in previous studies that have shown histological structure changes in the asymptomatic contralateral tendon [16, 59, 60]. Overall, the results of the present study align with evidence supporting the bilateral nature of tendinopathy and the involvement of CNS mechanisms.

### MS attributes difference with respect to healthy adults

During walking at natural and fast speeds, the twenty-eight participants in our study had relatively similar individualized EMG activation profiles and MS attributes (i.e., activation timing profiles and muscle weightings) (MS_1_ to MS_4_) compared to those previously reported in the literature [26, 32, 34, 61–63]. However, some MS attribute differences in comparison with healthy adults warrant attention. For MS_1_, the gluteus medius and vastus medialis weightings were lower in this study. For MS_4_, the biceps femoris and semitendinosus weightings were lower, whereas the tibialis anterior weighting was greater. These differences could be explained bilaterally by a decrease in hip and knee stability during stance and an increase in tibialis anterior co-contractions during terminal swing, which has been previously described among adults with unilateral acute pain in the calf muscles [30]. Such changes could be interpreted as an adaptation of the muscle activation pattern in response to the injured tendon or aetiological causative factor of AT.

### Increasing the walking speed did not alter GRF asymmetries and MS attributes differences between LL

The secondary objective was to verify if increasing the walking speed revealed additional differences in terms of GRF asymmetries and MS attributes between LL. The results only partially supported the hypothesis. Thus, increasing the walking speed from 1.3 m/s to 1.6 m/s resulted in higher peak GRF values The same phenomenon has been observed in healthy adults in previous studies [49, 56, 64, 65]. However, increased walking speed had limited effects on GRF and MS differences between LL. Also, the number of synergies merged to a greater extent at fast speed compared to natural speed, yet merged equally on both LL. An increase in some peak GRF values during fast speed is more likely associated with adjustments in the mechanical output of the MS rather than differences in the profile and activation timings.

### Translating evidence into tendon rehabilitation clinical practice

Current AT rehabilitation in clinical practice focuses predominantly on tendon loading, as it stimulates the physiological adaptation of the muscle-tendon complex [4, 7]. In clinical practice, eccentric plantarflexor strengthening exercises and heavy-slow plantarflexor resistance training has been shown to reduce pain and improve function among adults with AT [66–68]. Based on the high recurrence rate of AT and potential persistent motor changes following AT, current clinical practice may fail to adequately address contributing peripheral or central factors that may impact motor control. On one hand, CNS cortical representation, interhemispheric inhibition and motor cortex excitability may warrant additional attention during rehabilitation [69, 70]. Accordingly, tendon loading exercises that would be externally paced could be an interesting complement to rehabilitation protocols to best modulate tendon pain and CNS motor control [71]. On the other hand, focusing solely on the symptomatic AT during rehabilitation may attenuate the potential beneficial effects given potential tendon changes on the asymptomatic tendon. Rehabilitation protocols may therefore also need to involve the asymptomatic LL to prompt beneficial tendon integrity and CNS motor control adaptations over the long term [4, 69].

### Limitations

Some limitations associated with the present study require discussion. The relatively modest sample of participants (*n* = 28) with AT and the heterogeneity of the participants in terms of symptom duration and pain intensity may have reduced the statistical power and limited subgroup analysis. Also, the activity-specific muscle EMG amplitude normalization approach used may have limited the ability to compare peak EMG activation profile differences between conditions. As previously mentioned, the fact that the asymptomatic tendon was used as the comparator to assess the impact of AT may also have attenuated the magnitude of MS changes since both LL may have been affected in adults with unilateral AT [12, 58, 59]. Investigating adults with chronic AT and comparing them to healthy counterparts may have provided additional insights. As a mitigation strategy, supplementary materials present a cosine similarity analysis of the muscle weightings between our data and previously published normative data of healthy adults [34]. Caution remains advised if attempting to generalize the current findings with other tendons (e.g., patellar tendon, supraspinatus tendon), other walking speeds (e.g., slow speed, self-selected speed) [72], or other walking conditions (e.g., overground walking) [73]. Personal characteristics also known to influence walking (e.g., gender, height, leg length, body weight) would also need additional attention if within-subject comparisons were to be performed in the future. Finally, higher impact activity soliciting a greater amount of force or generating a higher pain level compared to walking, such as running and jumping, might have detected greater differences in peak GRF and MS attributes given the higher loading at the Achilles tendon [4, 11, 74].

## Conclusion

The presence of AT had limited effects on peak GRF and MS number, composition and temporal profiles between the symptomatic and asymptomatic LL for level treadmill walking at natural and fast speeds. Peripheral (i.e., changes in the asymptomatic tendon) or central adaptations (i.e., corticospinal neuroplastic changes) related to chronic unilateral AT may explain the preserved quasi-symmetric LL motor control during natural and fast walking among adults with chronic unilateral AT. Increasing LL muscular demand further (e.g., running, jumping) may have altered the ability of adults with chronic AT to modulate excitatory and inhibitory control of their LL muscles. The paradigm shift in current tendon-focused rehabilitation strategies deserves continued attention to best address corticospinal neuroplasticity adaptations.

## Supplementary Information

**Additional file 1.**

## Data Availability

The datasets used and analyzed in this study are available from the corresponding author upon reasonable request.

## Abbreviations

AT: Achilles tendinopathy
GRF: Ground reaction forces
MS: Muscle synergies
EMG: Electromyography
LL: Lower limb
CNS: Central nervous system
NNMF: Non-negative matrix factorization
VISA-A: Victorian Institute of Sport Assessment-Achilles
LEFS: Lower Extremity Functional Scale
SD: Standard deviation
LTF: Lateral thrust force
MIF: Medial impact force
HBF: Horizontal braking force
HPF: Horizontal propulsive force
VIF: Vertical impact force
MVP: Minimal vertical peak
VPF: Vertical propulsive force
VAF: Variance accounted for
RF: Rectus femoris
VM: Vastus medialis
TA: Tibialis anterior
MG: Medial gastrocnemius
SO: Soleus
ST: Semitendinosus
BF: Biceps femoris
GM: Gluteus medius
