# Supplementary material for: Unilateral symptomatic Achilles tendinopathy has limited effects on bilateral lower limb ground reaction force asymmetries and muscular synergy attributes when walking at natural and fast speeds
Source: J Foot Ankle Res. 2022 Sep 7;15:66. doi: 10.1186/s13047-022-00570-3 (PMC9450385; doi:10.1186/s13047-022-00570-3)
Supplement: Supplementary file 1 — Additional file 1. [file 13047_2022_570_MOESM1_ESM.docx]

# Annex 1

Cosine similarity was used to compare the average muscle weightings of each MS with averaged values of previously reported MS extracted using NNMF of the same eight muscles during gait for healthy individuals (Clark, Ting, Zajac, Neptune, & Kautz, 2010). Cosine similarity were calculated as the inner product of two muscle vectors representing the averaged muscle activation of the eight muscles for each walking condition.

Cosine similarities (CS) values between muscle synergies weightings of the current study and synergies previously reported in the literature among healthy participants. Bold values indicate the two highest CS value for each synergy.

| Clark  Synergy | Synergy C1 | | Synergy C2 | | Synergy C3 | | Synergy C4 | |
| --- | --- | --- | --- | --- | --- | --- | --- | --- |
|  | Sympt | Asympt | Sympt | Asympt | Sympt | Asympt | Sympt | Asympt |
|  | Natural Speed | | | | | | | |
| C1 | **0.960** | **0.948** | 0.388 | 0.429 | 0.663 | 0.760 | 0.498 | 0.572 |
| C2 | 0.319 | 0.319 | **0.993** | **0.983** | 0.281 | 0.281 | 0.241 | 0.234 |
| C3 | 0.713 | 0.735 | 0.295 | 0.362 | **0.958** | **0.927** | 0.576 | 0.601 |
| C4 | 0.379 | 0.521 | 0.191 | 0.206 | 0.518 | 0.486 | **0.935** | **0.928** |
|  | Fast Speed | | | | | | | |
| C1 | **0.953** | **0.936** | 0.379 | 0.418 | 0.666 | 0.734 | 0.564 | 0.571 |
| C2 | 0.321 | 0.310 | **0.992** | **0.983** | 0.310 | 0.299 | 0.227 | 0.258 |
| C3 | 0.751 | 0.717 | 0.292 | 0.354 | **0.972** | **0.937** | 0.606 | 0.653 |
| C4 | 0.518 | 0.621 | 0.184 | 0.203 | 0.417 | 0.499 | **0.937** | **0.905** |

**Reference**

Clark, D. J., Ting, L. H., Zajac, F. E., Neptune, R. R., & Kautz, S. A. (2010). Merging of healthy motor modules predicts reduced locomotor performance and muscle coordination complexity post-stroke. *J Neurophysiol, 103*(2), 844-857. doi:10.1152/jn.00825.2009
